# Assembling the puzzle of antimicrobial resistance in staphylococcal biofilms

**DOI:** 10.1080/22221751.2026.2627073

**Published:** 2026-02-03

**Authors:** Thuy Nguyen, David McGiffin, Bin Lou, Yao Sun, Changrui Qian, Xenia Kostoulias, Wenhong Zhang, Anton Y. Peleg, Yue Qu

**Affiliations:** a Infection Program, Department of Microbiology, Monash Biomedicine Discovery Institute, Monash University, Clayton, Australia; b Centre to Impact AMR, Monash University, Melbourne, Australia; c Department of Cardiothoracic Surgery, The Alfred and Monash University, Melbourne, Australia; d Critical Care Research Group, The Prince Charles Hospital, Brisbane, Australia; e Department of Laboratory Medicine, The First Affiliated Hospital, Zhejiang University School of Medicine, Hangzhou, People’s Republic of China; f Consortium for Infection and Innovation (CII), The First Affiliated Hospital of Wenzhou Medical University, Wenzhou, People’s Republic of China; g Department of Infectious Diseases, The Alfred Hospital and School of Translational Medicine, Monash University, Melbourne, Australia; h Department of Infectious Diseases, National Centre for Infectious Diseases of China, Huashan Hospital, Shanghai Medical College, Fudan University, Shanghai, People’s Republic of China

**Keywords:** Staphylococcal biofilms, antimicrobial resistance (AMR), penetration, low-cell metabolism, acidic pH, inoculum effects, biofilm killing

## Abstract

Multiple mechanisms underpinning biofilm antimicrobial resistance (AMR) have been studied individually. This study aimed to integrate these mechanisms, to understand their contributions to staphylococcal biofilm AMR, as a part of a whole, and to elucidate key hurdles hindering effective biofilm eradication by antimicrobial agents. Nine antibiotics were selected against microplate-based biofilms formed by *Staphylococcus aureus* ATCC 25923 and *Staphylococcus epidermidis* RP62A. Four mechanisms, including repressed bacterial metabolism, the barrier effect of the biofilm extracellular polymeric substances (EPS) matrix, the acidic inner-biofilm pH, and inoculum effects associated with high-cell-density biofilm growth were studied. The impact of individual mechanism on biofilm AMR was quantitated by determining the fold increase of concentration that allows antibiotics to overcome the mechanism. Antibiotic concentrations were then incrementally increased from minimum bactericidal concentration (MBC) to sequentially address all four mechanisms, ultimately aiming to kill at least 99.9% of biofilm cells. A simplified method was developed to evaluate the dependence of antibiotics on bacterial metabolic states for the lethality. Gentamicin, tobramycin and ciprofloxacin at 1024 µg/mL overcame all four mechanisms and successfully killed *S. aureus* ATCC 25923 biofilms by at least 3 log units. Ciprofloxacin at 1024 µg/mL effectively killed *S. epidermidis* RP62A biofilms. The contribution of each mechanism to biofilm AMR was strain- and drug-dependent, with low-cell metabolism being the most important factor. This study underscores the individual contributions of each mechanism to staphylococcal biofilm AMR and highlights the necessity of targeting all four mechanisms to achieve effective biofilm eradication.

## GRAPHICAL ABSTRACT

**Figure:**
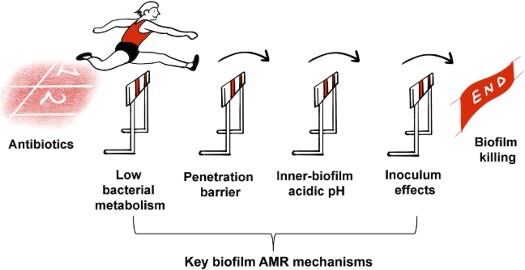

## Introduction

Microbial biofilms are known to cause many hospital-acquired medical device-related infections or other recurrent infections [1,2]. Clinical biofilms of diverse morphologies have been observed in patients with ventricular assist device driveline infections, recurrent vulvovaginal candidiasis, or extracorporeal membrane oxygenation (ECMO) cannula infections [3-5]. Although considerable effort has been spent to develop novel anti-biofilm strategies, very few have proven effective in large-scale clinical trials [6-8]. Prevention of biofilm formation is often a clinically preferred intervention due to the difficulty in eradicating established biofilms using chemical or physical measures. Current treatment guidelines and expert consensus statements for medical device – associated or biofilm-related infections, published by multiple medical societies, including those from the Centers for Disease Control and Prevention (CDC) on recurrent vulvovaginal candidiasis and the International Society for Heart and Lung Transplantation on mechanical circulatory support infections, primarily aim for long-term infection suppression and often fail to cure the infections [2,9-11]. A therapeutic arsenal that can be used to effectively eradicate infective biofilms is urgently needed.

Recent advances in chemical engineering, in particular nanofabrication techniques, have seen the development of many novel “anti-biofilm” drugs or drug-delivery systems [12]. Despite the accelerated development of novel anti-biofilm chemicals or drug-delivery systems, very few have reached the clinical application phase, partially due to the lack of a comprehensive understanding of antimicrobial resistance (AMR) of biofilms.

AMR of microbial biofilms and associated infections has been the topic of intensive research since the 1980s [13,14]. Numerous studies have been carried out to investigate mechanisms underpinning biofilm AMR, with the majority focusing on a single molecular or physiochemical mechanism [15,16]. Biofilm formation is a population phenotype and the associated AMR is known to be the result of the cumulative effect of multiple mechanisms [15,16]. Addressing a single mechanism or two may be inadequate to combat the stubborn biofilm AMR issues [15,16]. Indeed, experimental antimicrobials targeting only one or two biofilm AMR mechanisms, such as the quorum-sensing responses, have seen little clinical success. Our group has recently identified the low cell metabolic status as the central mechanism underlying biofilm AMR [17]. Other mechanisms, such as the presence of the biofilm extracellular polymeric substance (EPS) matrix that limits drug penetration, acidic inner biofilm pHs, and the inoculum effects associated with the high-density biofilm growth mode, have all been reported to play important roles in biofilm AMR [15,16,18-20]. Although individual mechanisms of biofilm AMR have been extensively studied, their contributions have yet to be compared and combined. It remains unknown which are the key hurdles that a novel antimicrobial agent needs to overcome in order to effectively eradicate microbial biofilms.

Lack of an integrative understanding of biofilm AMR mechanisms severely limits our ability to effectively treat biofilm-associated infections with weapons currently available in our antimicrobial arsenal. In this study, we aim to elucidate the contribution of the above-mentioned mechanisms, both individually and combined, to biofilm AMR, and we hope to establish screening rules that can guide chemical engineers to design, develop, and assess more effective anti-biofilm drugs.

## Materials and methods

### Methodological overview

**Figure 1.**
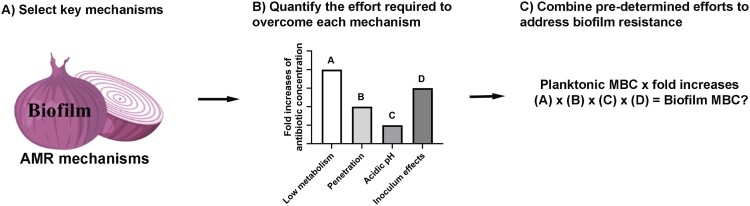
Methodological overview of this study. (A) Select four phenotypic biofilm AMR mechanisms representing or closely associated with most published molecular or chemical mechanisms. (B) Determine fold increases of antibiotic concentration required to overcome each of the four selected biofilm AMR mechanisms. (C) Add the fold increases in sequence to a therapeutic concentration of antibiotics until achieving successful biofilm killing. MBC, minimum bactericidal concentration.

The “reverse onion peeling strategy” was adopted to dissect layer by layer the AMR mechanisms of staphylococcal biofilms (Figure 1). Published single molecular and physiochemical mechanisms were reviewed; four mechanisms that either represent or have close association with these molecular or physiochemical mechanisms were selected for this study, including the low-metabolic states of biofilm cells [17], the biofilm EPS matrix barrier [20], the acidic inner-biofilm pH [21], and inoculum effects associated with the high-cell-density biofilm growth [19] (see Table S1). The impact of individual mechanisms on biofilm AMR was quantitated by determining the fold increase in antibiotic concentration required to overcome each of the four mechanisms. Fold increases in antibiotic concentrations were sequentially added to the Clinical & Laboratory Standards Institute (CLSI) minimum bactericidal concentration (MBC) to address the four mechanisms in order, until a concentration achieving a 3-log reduction in biofilm viability was reached, defined as the biofilm MBC. Such a method would allow us to evaluate the importance of any single mechanism on biofilm AMR, either independently or in combination.

### Microorganisms and antibiotics

Two biofilm-producing laboratory reference strains *Staphylococcus aureus* ATCC 25923 and *Staphylococcus epidermidis* RP62A and nine conventional antibiotics were selected for this study, including oxacillin, vancomycin, ciprofloxacin, rifampicin, and five aminoglycosides, gentamicin, kanamycin, tobramycin, streptomycin, and amikacin (Sigma, USA). Kanamycin and streptomycin have been historically used for *Staphylococcus* infections, and their current utility is limited by the widespread resistance of *Staphylococcus* spp. [22]. These two drugs were included to probe the impact of microorganisms’ intrinsic resistance on biofilm AMR and its individual mechanisms. The maximum concentration of antibiotics used in this study was 1024 µg/mL.

### Antimicrobial and anti-biofilm susceptibility testing

Antibiotic susceptibility testing for planktonic cells was carried out by assessing the minimum inhibitory concentrations (MIC) and MBCs, following the CLSI guideline M07-Ed12 [23]. For anti-biofilm susceptibility testing, staphylococcal biofilms were grown in 96-well microplates with tryptic soy broth (TSB, Oxoid, UK) overnight and treated with antibiotic solutions prepared in Muller-Hinton broth (MHB, Oxoid, UK) for 18 h at 37° C [24]. Anti-biofilm activities of antibiotics were determined by comparing the colony-forming-unit (CFU) before and after the treatment, and presented as log CFU reduction [25].

### Determining the dependence of antibiotic lethality on bacterial metabolic states

We simplified the comprehensive method published by Zheng *et al.* to evaluate the dependence of antibiotic lethality on bacterial metabolic states, by determining MBCs of antibiotics against planktonic cells grown in a nutrient-deprived growth medium [1% MHB + 99% phosphate buffered saline (PBS), MBC_low-metabolism_] [26]. The 1% MHB + 99% PBS medium provides minimal nutrients allowing bacterial cells to remain viable at a relatively low metabolic state [26]. The fold increase in antibiotic concentration required to overcome the low bacterial metabolism mechanism was calculated by dividing the CLSI-defined MBC by the MBC_low-metabolism_. The Log₂ (MBC_low-metabolism_/MBC) value was then calculated for each bacterium – antibiotic combination and used to classify antibiotics into three categories: 0–3, low dependence on bacterial metabolism; 4–7, moderate dependence; and 8–11, high dependence.

### Determining the impact of low inner-biofilm pHs on antibiotic activity against biofilms

The impact of acidic inner-biofilm pHs on antibiotic activity was assessed by comparing MICs and MBCs of antibiotics against planktonic cells in MHB pre-adjusted to pH = 5.5 and pH = 7.2. A pH of 5.5 represents the acidic inner-biofilm microenvironment and pH = 7.2 reflects that of the biofilm outer layer adjacent to the surrounding human components such as the blood or tissue fluids [21]. The fold increase of antibiotic concentrations required to overcome the impact of inner-biofilm pHs was calculated by dividing the MBC at pH = 5.5 (MBC_5.5_) by that at pH = 7.2 (MBC_7.2_).

### Determining the importance of inoculum effects on biofilm AMR

To assess the inoculum effects excluding low biofilm cell metabolism, MBC_low-metabolism_ of antibiotics was determined against planktonic cultures prepared at the CLSI-recommended testing density (∼1 × 10^6^ CFU/mL) and a higher density of ∼1 × 10^8^ CFU/mL respectively. The ratios of [MBC_low-metabolism_ (∼1 × 10^8^ CFU/mL) / MBC_low-metabolism_ (∼1 × 10^6^ CFU/mL)] represent the fold increase in antibiotic concentrations needed to overcome inoculum effects.

## Statistical analysis

All experiments were performed with at least three biological replicates. Differences in biofilm killing or log CFU reduction were analyzed, using one-way analysis of variance (ANOVA) or a non-parametric method, depending on data distribution. Statistical analysis was performed using the Minitab Statistical Software 16 for Windows (Minitab Ltd., Coventry, UK) with a significance level of 0.05.

## Results

### Addressing hurdle/mechanism 1: compromised antibiotic lethality against biofilm cells in low metabolic states

As successful treatment of biofilm-associated infections requires eradication of metabolically inactive biofilm-embedded cells, MBC_low-metabolism_ (as opposed to CLSI MBCs) was used as the starting concentration. These values were determined for all antibiotics against planktonic bacterial cells grown in nutrient-deprived conditions and are presented in Table 1. *S. aureus* ATCC 25923 and *S. epidermidis* RP62A cells had high MBC_low-metabolism_ of 1024 µg/mL for vancomycin and 256 µg/mL for oxacillin, 64-128 µg/mL for ciprofloxacin, and 1 µg/mL for rifampicin. Aminoglycosides in general showed good activity against metabolically inactive *S. aureus* ATCC 25923, supported by relatively low MBC_low-metabolism_ of 4-128 µg/mL. *S. epidermidis* RP62A is intrinsically resistant to most aminoglycosides and thus unsurprisingly had high MBC_low-metabolism_ (≥ 1024 μg/mL) for all five aminoglycosides. Notably, rifampicin was the only antibiotic with an MBC_low-metabolism_ below its serum-achievable concentration (30 µg/mL).

**Table 1.** Minimum bactericidal concentration against bacterial cells in low metabolic states (MBC_low-metabolism_) and categorization of antibiotics based on their dependence on cell metabolism for the lethality.

Microorganisms/Antibiotic	Van (µg/mL)	Oxa (µg/mL)	Cip (µg/mL)	Rif (µg/mL)	Gen (µg/mL)	Tob (µg/mL)	Stp (µg/mL)	Kan (µg/mL)	Amk (µg/mL)
***S. aureus* ATCC 25923**									
CLSI MBC	2	1	1	0.016	0.5	0.5	8	4	2
MBC_low-metabolism_	1024	256	128	1	4	2	128	32	16
**Fold change**	**512**	**256**	**128**	**64**	**8**	**4**	**16**	**8**	**8**
									
Log_2_ (MBC_low-metabolism_/MBC)	9	8	7	6	3	2	4	3	3
Metabolism-dependence of antibiotics for lethality*	High	High	Moderate	Moderate	Low	Low	Moderate	Low	Low
									
***S. epidermidis* RP62A**									
CLSI MBC	2	128	0.5	0.016	16	8	>128	>128	16
MBC_low-metabolism_	1024	256	64	1	>1024	1024	>1024	>1024	1024
**Fold change** ** **	**512**	**2**	**128**	**64**	**>64**	**128**	**NA**	**NA**	**64**
Log_2_ (MBC_low-metabolism_/MBC)	9	1	7	6	>6	7	NA	NA	6
Metabolism-dependence of antibiotics for lethality	High	NA	Moderate	Moderate	NA	NA	NA	NA	NA

Van, vancomycin; oxa, oxacillin; cip, ciprofloxacin; rif, rifampicin; gen, gentamicin; tob, tobramycin; stp, streptomycin; kan, kanamycin; amk, amikacin. *Metabolism-dependence of antibiotics for lethality was placed into different categories based on log_2_ (MBC_low-metabolism_/MBC): 0-3, low dependence; 4-7, moderate dependence; 8-11, high dependence. Antibiotics with higher metabolism-dependence for lethality have poorer activity against metabolically inactive bacterial cells. NA, not applicable due to intrinsic resistance of the bacterial strain to antibiotics. Results were calculated from three independent assays in technical triplicates.

We also assessed all nine conventional antibiotics for their dependence on bacterial metabolic states for lethality. These antibiotics were categorized into three groups based on their Log_2_ (MBC_low-metabolism_/MBC), with vancomycin in the high-dependence group, ciprofloxacin and rifampicin in the moderate category when treating both bacterial strains, oxacillin in the high-dependence category and most aminoglycosides in the low-dependence group for *S. aureus* ATCC 25923 (Table 1). Categorization of oxacillin and aminoglycosides for *S. epidermidis* RP62A was inapplicable owing to its intrinsic resistance to these antibiotics. None of the selected antibiotics at MBC_low-metabolism_ achieved greater than a 1-log reduction in staphylococcal biofilm cells (Figure 2A), highlighting the contribution of other mechanisms in biofilm AMR that require further investigation.

**← Figure 2.**
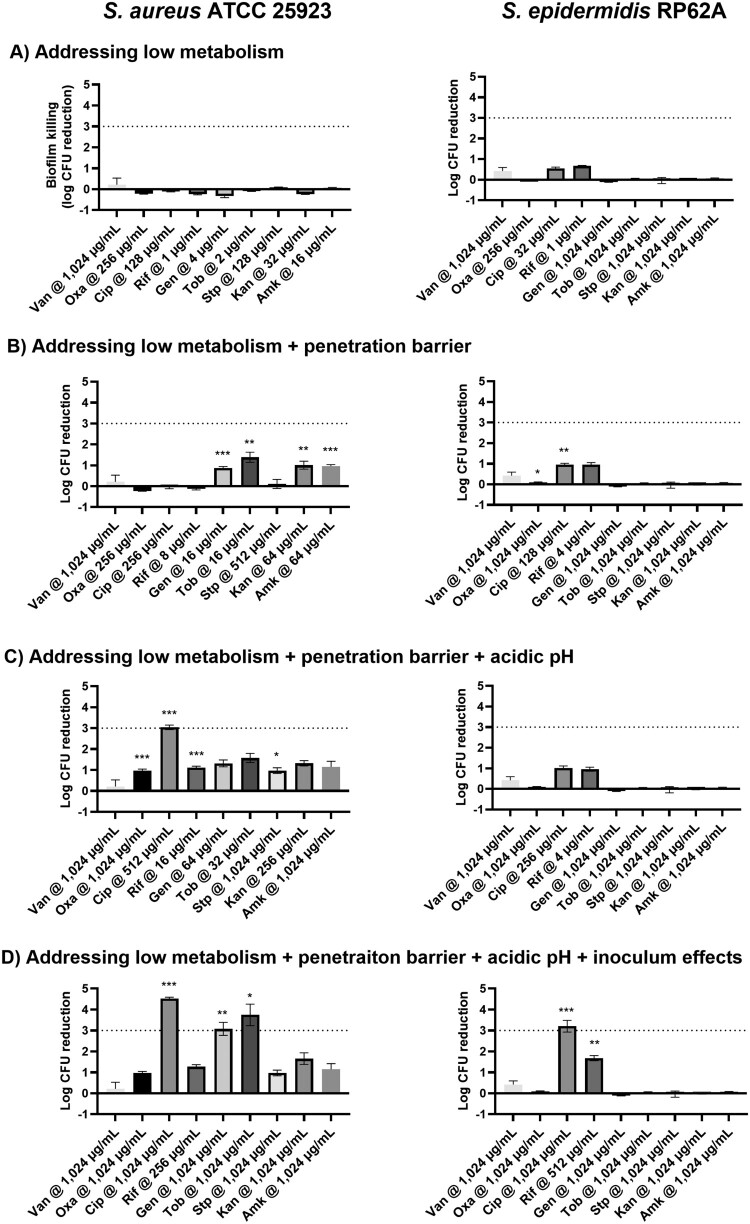
Killing of staphylococcal biofilms by antibiotics at concentrations that overcome single or multiple biofilm AMR mechanisms. (A) Antibiotics at concentrations needed to overcome the low cell metabolism mechanism, (B) Antibiotics at concentrations required for the low cell metabolism and biofilm extracellular polymeric substance (EPS) penetration barrier mechanisms. (C) Antibiotics at concentrations that were able to overcome the low cell metabolism, biofilm EPS penetration barrier and the inner-biofilm acidic pH mechanisms. (D) Antibiotics at concentrations that overcame the low cell-metabolism, biofilm EPS penetration barrier, inner-biofilm acidic pH, and inoculum effects. The data are shown as the average and standard error of 3–4 biological replicates. * indicates significant increases of biofilm killing when antibiotic concentrations were increased to overcome the current mechanism or hurdle. *, *P* < 0.05; **, *P* < 0.01; ***, *P* < 0.001.

### Addressing hurdle/mechanism 2: biofilm EPS matrix as an antibiotic penetration barrier

Another well-established biofilm AMR mechanism is the barrier effect of the biofilm EPS matrix, though this effect appears to be strain- and antibiotic-specific and possibly non-essential [20,27]. Our very recent study quantitatively evaluated the penetration of these antibiotics through colony biofilms formed by *S. aureus* ATCC 25923 and *S. epidermidis* RP62A (Detailed methods and results can be found in [20]). In the current study, we increased antibiotic concentrations to overcome the penetration barrier of biofilm EPS, by multiplying the MBC_low-metabolism_ with the reciprocal of the antibiotic biofilm penetration ratios. The fold increases of concentration theoretically allow sufficient amounts of antibiotics to reach biofilm-embedded cells and kill metabolically inactive cells. Antibiotics at concentrations that overcame both the low cell metabolism and the EPS penetration barrier, however, only killed biofilm cells at a minimum level, with CFU log reductions ranging from -0.23 ± 0.02 to 1.38 ± 0.49 (Figure 2B). These findings underscore the need to identify and target additional resistance mechanisms.

### Addressing hurdle/mechanism 3: drug-specific impact of acidic inner-biofilm pHs on antibiotics

The negative effect of acidic pH on the activity of certain antibiotics has been reported [28]. Biofilms represent a complex and dynamic infection microenvironment, with their surface areas presenting a pH similar to the neighbouring human body fluid or the blood, and the inner-biofilm microenvironment often demonstrating an acidic pH. We tested the impact of an acidic pH (5.5) on the activities of nine antibiotics and observed an adverse effect on aminoglycosides, with MICs and MBCs increasing by an average of 5.28-fold (ranges, 2–8-fold for MICs and 2–16-fold for MBCs) against *S. aureus* ATCC 25923, and by an average of 4-fold (range, 1 – >16-fold) and 12.7-fold (range, >2–16-fold), respectively, against *S. epidermidis* RP62A (Table 2). Acidic pH had little or no effect (1/4-2-fold) on vancomycin, ciprofloxacin and rifampicin MICs and MBCs when tested against the two staphylococcal strains (Table 2). The fold increase of antibiotic concentrations needed to overcome the acid inner-biofilm pHs was calculated, and the concentration of antibiotics was increased accordingly to compensate for the negative effect of acidic pH on antibiotic activities and to simultaneously address the three mechanisms (Table 4). Evident biofilm killing was observed for oxacillin, ciprofloxacin, rifampicin, gentamicin, tobramycin, kanamycin, and amikacin against *S. aureus* ATCC 25923 (>1-log reduction; Figure 2C), as well as for ciprofloxacin and rifampicin against *S. epidermidis* RP62A (>1-log reduction). The biofilm killing of most antibiotics, however, did not reach the desired three-log reduction (Figure 2C).

**Table 2.** Impact of acidic pHs of the inner-biofilm microenvironment on antibiotic activities.

Microorganisms/Antibiotic		Van (µg/mL)	Oxa (µg/mL)	Cip (µg/mL)	Rif (µg/mL)	Gen (µg/mL)	Tob (µg/mL)	Stp (µg/mL)	Kan (µg/mL)	Amk (µg/mL)
***S. aureus* ATCC 25923**										
** **	MIC_7.2_*	1	0.25	0.5	0.008	0.25	0.25	8	2	2
** **	MIC_5.5_	1	0.25	1	0.008	2	0.5	64	8	16
** **	Fold change	1	1	2	1	8	2	8	4	8
** **	MBC_7.2_	2	1	1	0.016	0.5	0.5	8	4	2
** **	MBC_5.5_	2	4	2	0.03	2	1	64	16	32
** **	**Fold change**	**1**	**4**	**2**	**2**	**4**	**2**	**8**	**4**	**16**
***S. epidermidis* RP62A**										
** **	MIC_7.2_	2	64	0.125	0.008	8	8	>128	>128	8
	MIC_5.5_	2	64	0.25	0.004	>128	8	>128	>128	32
	Fold change	1	1	2	1/2	>16	1	NA	NA	4
	MBC_7.2_	2	128	0.5	0.016	16	8	>128	>128	16
	MBC_5.5_	2	128	1.0	0.004	>128	128	>128	>128	256
	**Fold change**	**1**	**1**	**2**	**1/4**	**>8**	**16**	**NA**	**NA**	**16**

MIC, minimum inhibitory concentration. *MIC_7.2_, MIC at pH = 7.2. MIC_5.5_, MIC at pH = 5.5. MBC, minimum bactericidal concentration. Van, vancomycin; oxa, oxacillin; cip, ciprofloxacin; rif, rifampicin; gen, gentamicin; tob, tobramycin; stp, streptomycin; kan, kanamycin; amk, amikacin. NA, not applicable due to intrinsic resistance of the bacterial strain to antibiotics. Shown are results of three biological replicates.

### Determining hurdle/mechanism 4: inoculum effects associated with high-cell-density biofilm growth mode

Inoculum effects caused by high-cell-density growth are a well-established characteristic of microbial biofilms and are known to contribute to biofilm AMR [19,29]. We assessed the impact of inoculum effects, excluding the low-cell metabolism, on antibiotic activities. Comparison of MBC_low-metabolism_ at different cell densities suggested that high inoculum has a significant impact on the studied antibiotics in the context of bacterial killing. The MBC_low-metabolism_ of aminoglycosides increased by >8-32 fold when the densities of *S. aureus* cultures were increased from 1 × 10^6^ CFU/mL to 1 × 10^8^ CFU/mL; that of oxacillin and rifampicin increased by >4 fold and 16-fold, respectively (Table 3). Ciprofloxacin showed good tolerance against the high inoculum effect, with only two- to four-fold changes observed for both staphylococcal strains (Table 3).

**Table 3.** Effects of high bacterial inoculum on antibiotic activities excluding the low cell metabolism.

	Van (µg/mL)	Oxa (µg/mL)	Cip (µg/mL)	Rif (µg/mL)	Gen (µg/mL)	Tob (µg/mL)	Stp (µg/mL)	Kan (µg/mL)	Amk (µg/mL)
***S. aureus* ATCC 25923**									
MBC_low-metabolism_ @ 1 × 10^6^ CFU/mL	1024	256	128	1	4	2	128	32	16
MBC_low-metabolism_ @ 1 × 10^8^ CFU/mL	>1024	>1024	256	16	64	64	>1024	512	512
**Fold change**	**>1**	**>4**	**2**	**16**	**16**	**32**	**>8**	**16**	**32**
***S. epidermidis* RP62A**									
MBC_low-metabolism_ @ 1 × 10^6^ CFU/mL	1024	256	64	1	>1024	1024	>1024	>1024	1024
MBC_low-metabolism_ @ 1 × 10^8^ CFU/mL	>1024	>1024	256	128	>1024	>1024	>1024	>1024	>1024
**Fold change**	**>1**	**>4**	**4**	**128**	**NA**	**NA**	**NA**	**NA**	**NA**

The microbial inoculum effect was quantitatively determined by comparing MBC_low-metabolism_ against bacterial suspensions at a high inoculum (∼1 × 10^8^ CFU/mL) and the standard inoculum (∼ 1 × 10^6^ CFU/mL). MBC_low-metabolism_ was used to exclude the impact of low bacterial metabolic states, which was studied elsewhere (see table 1). Van, vancomycin; oxa, oxacillin; cip, ciprofloxacin; rif, rifampicin; gen, gentamicin; tob, tobramycin; stp, streptomycin; kan, kanamycin; amk, amikacin. NA, not applicable due to intrinsic resistance of the bacterial strain to antibiotics. Three biological repeats in technical triplicates were carried out.

### Addressing all four hurdles/mechanisms and sequentially overcoming low cell metabolism, penetration, acidic pH, and inoculum effects to effectively kill biofilm cells

We successively increased concentrations of antibiotics to a level that adequately overcame all four biofilm AMR mechanisms, including low cell metabolism, penetration barrier, acidic pH, and high inoculum effects (Table 4). At the accrued concentration, ciprofloxacin killed *S. aureus* ATCC 25923 biofilms by 4.52 ± 0.12 logs and *S. epidermidis* RP62A biofilms by 3.20 ± 0.21 logs (Figure 2D). Gentamicin and tobramycin at the desired biofilm MBC of 1024 µg/mL also effectively kill biofilms formed by *S. aureus* ATCC 25923, reducing biofilm CFUs by 3.08 ± 0.54 and 3.74 ± 0.89 log units. Interestingly, overnight exposure of *S. aureus* ATCC 25923 biofilms to rifampicin at 256 µg/mL and *S. epidermidis* RP62A biofilms to rifampicin at 512 µg/mL only resulted in 1.27 ± 0.16 and 1.68 ± 0.21 log reductions of biofilm cells. Oxacillin and vancomycin at 1024 µg/mL failed to overcome all four mechanisms and, as expected, were unable to kill staphylococcal biofilm cells by three log units.

**Table 4.** Stepwise increasing antibiotic concentrations to overcome multiple biofilm antimicrobial resistance mechanisms.

*S. aureus* ATCC 25923 biofilm & hurdles	Van (µg/mL)	Oxa (µg/mL)	Cip (µg/mL)	Rif (µg/mL)	Gen (µg/mL)	Tob (µg/mL)	Stp (µg/mL)	Kan (µg/mL)	Amk (µg/mL)
CLSI MBC	2	1	1	0.016	0.5	0.5	8	4	2
1: Low metabolism (fold change)	512	256	128	64	8	4	16	8	8
Concentrations to address mechanism 1	1024	256	128	1	4	2	128	32	16
2: Penetration (%) *	Poor ^#^	100 ± 14	87 ± 11	19 ± 5	48 ± 0	18 ± 0	48 ± 18	82 ± 15	49 ± 0
(fold change)	NA	1	2	8	4	8	4	2	4
Addressing mechanisms 1 + 2	>1024	256	256	8	16	16	512	64	64
3: Acidic pH (fold change)	1	4	2	2	4	2	8	4	16
Addressing mechanisms 1 + 2 + 3	>1024	1024	512	16	64	32	>1024	256	1024
4: Inoculum effects (fold change)	>1	>4	2	16	16	32	>8	16	32
Addressing mechanisms 1 + 2 + 3 + 4	>1024	>1024	1024	256	1024	1024	>1024	>1024	>1024
***S. epidermidis* RP62A biofilm & hurdles**	**Van (µg/mL)**	**Oxa (µg/mL)**	**Cip (µg/mL)**	**Rif (µg/mL)**	**Gen (µg/mL)**	**Tob (µg/mL)**	**Stp (µg/mL)**	**Kan (µg/mL)**	**Amk (µg/mL)**
CLSI MBC	2	128	0.5	0.016	16	8	>128	>128	16
1: Low metabolism (fold change)	512	2	128	64	>64	128	NA	NA	64
Addressing mechanism 1	1024	256	64	1	>1024	1024	>1024	>1024	1024
2: Penetration (%) *	Poor	67 ± 4	69 ± 6	25 ± 3	54 ± 0	36 ± 14	NA	NA	38 ± 3
(fold change)	NA	2	2	4	2	4	NA	NA	4
Addressing mechanisms 1 + 2	>1024	512	128	4	>1024	>1024	>1024	>1024	>1024
3: Acidic pH (fold change)	1	1	2	1^#^	>8	16	NA	NA	16
Addressing mechanisms 1 + 2 + 3	>1024	512	256	4	>1024	>1024	>1024	>1024	>1024
4: Inoculum effects (fold change)	>1	>4	4	128	NA	NA	NA	NA	NA
Addressing mechanisms 1 + 2 + 3 + 4	>1024	>1024	1024	512	>1024	>1024	>1024	>1024	>1024

*Antibiotic biofilm penetration ratios refer to our recently published study [20]. Van, vancomycin; oxa, oxacillin; cip, ciprofloxacin; rif, rifampicin; gen, gentamicin; tob, tobramycin; stp, streptomycin; kan, kanamycin; amk, amikacin. Antibiotic concentrations were calculated by dividing MBC_low-metabolism_ by the mean penetration ratio and rounded upwards to the next concentration. Numbers in bold indicate concentrations of antibiotics that overcome all four mechanisms and are still within the experimental limit (1024 µg/mL). ^#^, fold change of 1 was used instead of ¼ (see table 2) to address both inner and outer biofilm cells. NA, not applicable due to intrinsic resistance of the bacterial strain to antibiotics.

### Comparing relative weights of different mechanisms contributing to biofilm AMR

We further evaluated the relative contribution of individual mechanisms to biofilm AMR, by comparing the log_2_ of fold increases in antibiotic concentrations that were required to overcome each mechanism. Low cell metabolism appeared to be the most important mechanism underlying biofilm-associated AMR, evidenced by the relatively greater fold increase in antibiotic concentration needed to overcome it, followed by inoculum effects (Table 5). This finding is in line with our recent statement highlighting the central role of the low bacterial metabolic state in biofilm AMR [17]. The importance of biofilm EPS penetration barrier and acidic inner-biofilm pH seems to be less substantial and strain – and antibiotic – specific for biofilm AMR.

**Table 5.** Relative contribution of individual mechanisms to biofilm antimicrobial resistance (AMR)

Biofilms and AMR mechanisms	Log 2 (fold change) of antibiotic concentrations to overcome different biofilm AMR mechanisms								
***S. aureus* ATCC 25923**	**Van**	**Oxa**	**Cip**	**Rif**	**Gen**	**Tob**	**Stp**	**Kan**	**Amk**
*Hurdle 1: Low metabolism*	9	8	7	6	3	2	4	3	3
*Hurdle 2: Penetration*	NA	0	1	3	2	3	2	1	2
*Hurdle 3: Acidic pH*	0	2	1	1	2	1	3	2	4
*Hurdle 4: Inoculum effects*	>0	>2	1	4	4	5	>3	4	5
***S. epidermidis* RP62A**	**Van**	**Oxa**	**Cip**	**Rif**	**Gen**	**Tob**	**Stp**	**Kan**	**Amk**
*Hurdle 1: Low metabolism*	9	1	7	6	>6	7	NA	NA	6
*Hurdle 2: Penetration*	NA	1	1	2	1	2	NA	NA	2
*Hurdle 3: Acidic pH*	0	0	1	−2	>3	4	NA	NA	4
*Hurdle 4: Inoculum effects*	>0	>2	2	7	NA	NA	NA	NA	NA

Van, vancomycin; oxa, oxacillin; cip, ciprofloxacin; rif, rifampicin; gen, gentamicin; tob, tobramycin; stp, streptomycin; kan, kanamycin; amk, amikacin.

NA, not applicable due to intrinsic resistance of the bacterial strain to antibiotics.

## Discussion

Biofilm AMR phenotypes and underlying mechanisms have been extensively studied in the past decades. While significant progress has been made in exploring individual mechanisms contributing to biofilm AMR, an understanding of how these mechanisms integrally contribute to biofilm AMR is lacking. A consensus has been reached within the biofilm research community that a single mechanism cannot explain the high level of biofilm AMR, and multiple mechanisms collaboratively underpin this phenotype [15,16].

Methodologically, we developed a pipeline of microbiological assays that allows diagnostic laboratories or engineering laboratories to assess how well an antimicrobial agent overcomes key biofilm AMR mechanisms. A highlight is the optimized and simplified method that can be used to evaluate the dependence of antibiotics on bacterial metabolism for lethality. In comparison with the original method published by Zheng *et al.* [26], the current method is much less labour-intensive and time-consuming; however, still offers similarly valuable information that can guide clinicians to select the most effective antibiotics for biofilm infections characterized by the presence of metabolically inactive bacterial cells.

This study comprehensively re-evaluated different mechanisms underlying staphylococcal biofilm AMR using a panel of conventional antibiotics. Starting with the CLSI MBC as the baseline concentration, antibiotic concentrations were incrementally increased to overcome four important mechanisms that contribute to biofilm AMR: Low metabolism of biofilm-embedded cells, biofilm EPS matrix as antibiotic penetration barriers, acidic pH of the inner biofilm microenvironment that compromises antibiotic activities, and inoculum effects associated with high-cell-density biofilm growth mode. The cumulative concentrations aimed to reach the biofilm MBC, defined as killing staphylococcal biofilm cells by at least 3-log units. This endpoint was successfully achieved when ciprofloxacin, tobramycin, and gentamicin were used for *S. aureus* ATCC 25923 and ciprofloxacin for *S. epidermidis* RP62A; other antibiotics at the highest experimental concentration (1024 µg/mL) were unable to address all four mechanisms and failed to effectively kill biofilm cells.

Two aminoglycosides, gentamicin and tobramycin, at concentrations much higher than their serum-achievable concentrations, have been frequently used as topical therapies for biofilm-related orthopedic or cardiac implant-related infections and in some reports, were able to cure infections without the need for replacement of infected devices [30,31]. The low dependence of aminoglycosides on bacterial metabolism supports their clinical efficacy. Although the antibiofilm activities of aminoglycosides are significantly affected by inoculum effects and acidic inner-biofilm pHs, these hurdles can be overcome by increasing the antibiotic concentration to a level that is effective against biofilms and biocompatible to human cells when used locally [31]. Oxacillin and vancomycin are first-line antibiotics in clinical settings for methicillin-sensitive and methicillin-resistant staphylococcal infections. Consistent with what was found in the current study, these two antibiotics have been proven to be ineffective for staphylococcal biofilms by numerous *in vitro* and *in vivo* studies [19,24]. Although oxacillin can readily penetrate through biofilm formed by *S. aureus* ATCC 25923, its lethality is highly dependent on bacterial metabolism and further compromised by inoculum effects of biofilms. Vancomycin also suffers from these same inadequacies as oxacillin and an additional biofilm AMR mechanism that is the inability to penetrate through biofilm EPS [20]. Ciprofloxacin and rifampicin have been used as the last-resort antibiotics for implant-related staphylococcal infections, often in combination with other antibiotics. Although ciprofloxacin exhibits a moderate dependence on bacterial metabolic activity, it showed excellent performance against the other three resistance mechanisms. Topical application of ciprofloxacin and moxifloxacin has been shown to effectively eradicate *S. aureus* in experimental osteomyelitis models [32,33]. However, the clinical use of high-concentration fluoroquinolones applied locally in orthopedic settings should be carefully validated and rationalized, given the potential for systemic absorption and the risk of rare neurological complications [33]. Rifampicin is a controversial antibiotic. It is the only drug that can overcome most biofilm AMR mechanisms at relatively low concentrations. However, its antibiofilm efficacy as a single agent was not seen in the current study, possibly due the rapid development of rifampicin-resistant strains or the presence of *Agr*-controlled rifampicin efflux system in biofilms formed by *S. aureus* ATCC 25923 and *S. epidermidis* RP62A [34]. A future study is warranted.

We also compared the relative contribution of individual mechanisms to biofilm AMR. We previously identified the low metabolism of biofilm cells as the central mechanism of biofilm AMR [17]. Low metabolism of biofilm cells has a universal impact on the activities of all tested antibiotics and appeared to have greater impact on staphylococcal biofilm AMR against vancomycin, ciprofloxacin, rifampicin and oxacillin. Other mechanisms, including the biofilm penetration barrier, acidic inner-biofilm microenvironment, and inoculum effects, had relatively minor and drug- and microorganism-specific impacts on biofilm AMR.

There are limitations to this study. The four mechanisms examined in this study do not provide a comprehensive explanation of other mechanisms that may be less critical but still make a substantial contribution to biofilm AMR. Other mechanisms not addressed include polymicrobial interactions [15,35], and those causing genotypic resistance of biofilm cells, including horizontal gene transfer [36], transient resistance [16,35], spontaneous mutagenesis, multi-drug efflux [16,35], altered drug target [35], AMR plasmid persistence [15] and acquisition of point mutation [15]. Clinically, the contribution of these genetic determinants to biofilm resistance is thought to be minor relative to the widespread and significant impact of phenotypic biofilm tolerance [37]. Methodologically, we only assessed biofilm killing to a 3-log reduction level in the current study and did not study the importance of persister cells. Experimental evidence has been provided in our very recent study to support the association between low cell metabolism and the presence of persister cells [17]. Treating staphylococcal biofilms with low-or-moderate-cell-metabolism-dependent antibiotics such as gentamicin or ciprofloxacin for a prolonged period may further kill persister cells residing in biofilms [17,38]. Another limitation is the small number of bacterial strains used in this study. We only used two laboratory reference staphylococcal strains that are well-known biofilm producers. Other isolates may form biofilms with differing matrix compositions or varying cell densities; contribution of the four mechanisms towards AMR of biofilms formed by other strains may differ significantly. The current study also suffers from its *in vitro* nature. Clinical biofilms differ from microplate-based *in vitro* biofilms in their morphologies, structural characteristics and possibly have even higher AMR in the complex infection microenvironment.

In conclusion, this study comprehensively and integrally evaluated multiple mechanisms underpinning biofilm AMR. Four mechanisms, including low metabolism of biofilm-embedded cells, penetration barriers, acidic inner-biofilm pHs, and inoculum effects associated with high-cell-density biofilm growth, were all critical for biofilm AMR and need to be simultaneously addressed for effective anti-biofilm therapies. We also provided detailed methodologies that allow microbiologists and chemical engineers to assess the effectiveness of conventional or newly developed drugs against individual and combined biofilm AMR mechanisms. Newer antibiotics that have been clinically approved for the treatment of *Staphylococcus* infections, such as daptomycin, telavancin, and dalbavancin, should be investigated in future studies for their activity against biofilms and their capacity to overcome individual biofilm AMR mechanisms.

## Supplementary Material

Supplementary materials R1.pdf
