# Supplementary material for: Assembling the puzzle of antimicrobial resistance in staphylococcal biofilms
Source: Emerg Microbes Infect. 2026 Feb 3;15(1):2627073. doi: 10.1080/22221751.2026.2627073 (PMC12903938; doi:10.1080/22221751.2026.2627073)
Supplement: Supplementary materials R1.pdf [file TEMI_A_2627073_SM2779.pdf]

Table S1. Four biofilm AMR mechanisms selected for this study and their association with other published mechanisms

**Mechanisms selected for this study**				
1) Low metabolic states of biofilm cells[1]	2) EPS matrix as a penetration barrier[2]	3) Inner-biofilm acidic pH	4) Inoculum effects[3]	5) Other mechanisms
**Associated molecular or physicochemical mechanisms published by others**				
Altered microenvironment<br>• Nutrient gradient [4]<br>• Hypoxia[4,5]<br>• Accumulated metabolic byproducts[4]<br><br>Physiological and metabolic heterogenicity[4,5]<br><br>Reduced growth rate[4,5]<br>• Viable-but-nonculturable cells[6]<br>• Persister cells[4,5]	Physical barrier<br>• Polysaccharide[4,5,7]<br>• eDNA[4,5,7]<br>• Electrical interactions[5,7]<br><br>Chemical barrier<br>• Antibiotic-hydrolyzing enzymes [4,5]<br><br>Biological barrier<br>• Filamentous phages [4,5]<br><br>Impaired penetration [5]	Low pH of inner-biofilm microenvironment[8]<br><br>Heterotrophic metabolism[6]	Quorum-sensing responses[4,7]<br><br>Adaptative stress responses[4,5]<br>• The general stress RpoS response[5]<br>• Stringent responses[4,5]<br>• The SOS response[5]<br>• The oxidative stress response[4]<br><br>Horizontal gene transfer[9]<br><br>Persister cells[4,5]	Polymicrobial interactions[4,7]<br><br>Genetical biofilm resistance<br>• Transient resistance[4,5,7]<br>• AMR plasmid[7]<br>• Membrane vesicles[5]<br>• Spontaneous mutagenesis<br>• Acquisition of point mutation[7]<br>• Multi-drug efflux[4,5,9]<br>• Altered drug target[4,9]

References

[1] Sun Y, McGiffin D, Ye L, et al. Low bacterial metabolism as a central mechanism of antimicrobial resistance and potential therapeutic target of staphylococcal biofilms in ventricular assist device driveline infections. Journal of Antimicrobial Chemotherapy. 2025;80(11):2923-2933.

[2] Lou B, McGiffin D, Nguyen T, et al. Accurate quantitation of antibiotic penetration through staphylococcal biofilms. Biofilm 2025;10:100316.

[3] Qu Y, Daley AJ, Istivan TS, et al. Densely adherent growth mode, rather than extracellular polymer substance matrix build-up ability, contributes to high resistance of Staphylococcus epidermidis biofilms to antibiotics. J Antimicrob Chemother. 2010;65(7):1405-11.

[4] Hall CW, Mah TF. Molecular mechanisms of biofilm-based antibiotic resistance and tolerance in pathogenic bacteria. FEMS Microbiol Rev. 2017;41(3):276-301.

[5] Ciofu O, Moser C, Jensen PØ, et al. Tolerance and resistance of microbial biofilms. Nature Reviews Microbiology. 2022;20(10):621-635.

[6] Flemming HC, Wingender J, Szewzyk U, et al. Biofilms: an emergent form of bacterial life. Nat Rev Microbiol. 2016;14(9):563-75.

[7] Liu HY, Prentice EL, Webber MA. Mechanisms of antimicrobial resistance in biofilms. npj Antimicrobials and Resistance. 2024;2(1):27.

[8] Behbahani SB, Kiridena SD, Wijayaratna UN, et al. pH variation in medical implant biofilms: Causes, measurements, and its implications for antibiotic resistance. Front Microbiol. 2022;13:1028560.

[9] Sharma D, Misba L, Khan AU. Antibiotics versus biofilm: an emerging battleground in microbial communities. Antimicrob Resist Infect Control. 2019;8:76.
